# Disability and its determinants among individuals with onchocerciasis in Southeast Nigeria: a cross-sectional study

**DOI:** 10.11604/pamj.2022.42.249.33879

**Published:** 2022-08-03

**Authors:** Adah Emmanuel Otache, Ifeyinwa Lilian Ezenwosu, Edmund Ndudi Ossai, Elias Chikee Aniwada, Stephen Obekpa Abah, Benjamin Chudi Uzochukwu

**Affiliations:** 1 Department of Community Medicine, University of Nigeria Teaching Hospital, Enugu State, Nigeria,; 2 Department of Community Medicine, Federal Medical Center, Makurdi, Benue State, Nigeria,; 3 Department of Community Medicine, College of Health Sciences, Ebonyi State University, Abakaliki, Nigeria,; 4 Department of Community Medicine, Federal University of Health Sciences Otukpo, Benue State, Nigeria

**Keywords:** Disability, onchocerciasis, neglected tropical diseases

## Abstract

**Introduction:**

although onchocerciasis is rarely a life-threatening disease, it causes chronic morbidity which ultimately leads to disability due to late detection and treatment of the infected people. Disability in Onchocerciasis results in activity limitation and social exclusion of the affected individuals through stigma. This study aimed at assessing the level of disability and its determinants among persons with onchocerciasis.

**Methods:**

multi-stage sampling technique was used to recruit 340 respondents from the randomly selected wards. Disability was assessed using WHO Disability Assessment Schedule 2.0 (WHODAS 2.0) tool. Chi-square test and multivariate analysis using binary logistic regression were used to determine factors associated with disability. The level of statistical significance was determined by a p-value of < 0.05.

**Results:**

the highest proportion (35.6%) of the respondents was 55 years and above with a mean age of 46.8±17.5. About three-quarters (76.2%) of them had some form of disability and the disability domains mainly affected were participation in community activities (61.8%) and getting along with other people (54.1%) while the least affected were self-care (26.2%) and life activities including domestic responsibilities (45.0%). The Predictor of disability included being ≥48 years old, (AOR=0.2; 95%CI: 0.1-0.4).

**Conclusion:**

most people with onchocerciasis experience some form of disability and the major determinant for disability is being in the older age group. Thus, there is a need for stakeholders in the onchocerciasis control program to formulate and implement disability screening programs in the communities for early detection of onchocerciasis-associated disability, especially among the older age group.

## Introduction

Onchocerciasis is a neglected tropical disease of major public health importance that is caused by *Onchocerca volvulus*, a filarial parasite transmitted by the bite of an infected insect vector (black fly) [1]. It affects people especially farmers who live and work around fast-flowing rivers with rocky vegetation which serves as the breeding environment for the vector [2] Onchocerciasis occurs mainly in the tropical regions of the world where more than 99% of the infected people reside [1]. Although small foci of the disease exist in Latin America and Yemen, the majority occur in 31 African countries including Nigeria which accounts for about 40% of cases [3]. In sub-Saharan Africa, an estimated 120 million people are at risk of contracting the disease, and 300,000 cases of irreversible blindness have been reported in about 37 million infected people [4].

The clinical manifestation of Onchocerciasis involves two main regions; dermal and occular regions with occasional involvement of lymph nodes, peripheral nerves and body fluids in complicated infections [5]. The skin lesions consist of intense itching, papular onchodermatitis, palpable nodules, hyperpigmentation, depigmentation (lizard skin) usually on the anterior lower leg and skin atrophy [4]. Occular manifestation varies from visual impairment to permanent blindness. In long-standing infections, the lymph nodes are involved resulting in swollen lymph glands, lymphadenitis and hanging groin [5]. Although the clinical features of onchocerciasis are not life-threatening, they can cause visual and physical impairments which lead to chronic suffering and disability in the affected people [6].

Disability is any health condition that impairs, interferes with, or limits a person´s ability to engage in certain tasks or actions which may lead to restriction in participating in community activities and interactions [6]. Approximately 1.5 million disability-adjusted life-years (DALYs) are being lost each year due to Onchocerciasis, 40% of these DALYs lost are due to visual impairment and 60% to the severe itching associated with skin lesions [7]. People with disabilities due to physical or visual impairments attributed to onchocerciasis face several challenges such as low attainment of educational levels, loss of productivity time, unemployment, increased health care cost, poverty and poor general well-being [8]. Some may be stigmatized by family or community members due to fear of the disease being contagious and this can worsen the mental health of the affected individual leading to low self-esteem, anxiety, depression and ultimately reducing life expectancy [6, 8].

Also, family members of the affected individuals are stigmatized, they may drop out of school to attend to the needs of the disabled individual and in some situations, disability can lead to marital conflicts [6]. Thus, early detection of disability in onchocerciasis is important to minimize its negative impact on the infected individuals, their family and society. Furthermore, in individuals with other neglected tropical diseases, environmental and personal factors such as support from families, socioeconomic status, and age, have been noted to affect the level of the disability [9]. There is a need to identify the factors affecting disability in onchocerciasis for better formulation and implementation of the strategies that will address them to improve the quality of life of the infected people, especially in Southeast Nigeria where the disease is endemic [10]. This study aimed to assess the level of disability and its determinants among persons with onchocerciasis in Southeast Nigeria.

## Methods

**Study area:** the study was done in Enugu State, Southeast Nigeria. The State consists of 17 local government areas (LGAs) and several fast-flowing rivers that are highly oxygenated which promotes the breeding of blackflies in the State [11]. More than 50% of the people residing in the State are rural dwellers´ with the majority being farmers [11]. Also, some people are traders and government workers, especially in the urban areas of the State. Community-Directed Treatment with Ivermectin strategy by African Programme for Onchocerciasis Control (APOC) was implemented in the State in 1999. Since then, ivermectin is administered orally at a dose of 150µg/kg yearly to all the community members in the State by the community-directed distributors as prophylaxis for onchocerciasis [11].

**Study design:** this was a community-based, cross-sectional analytical study conducted between September 2019 and November 2019 in Enugu State, Southeast Nigeria.

**Study population:** adults aged 18 years and above with onchocerciasis or have complications from the disease who were identified through physical observations by the health care workers in the onchocerciasis unit of the State Ministry of Health. However, critically ill patients who cannot talk, persons with other chronic diseases e.g. diabetics, and individuals with co-morbidity with other NTDs like lymphatic filariasis, etc. were excluded from the study.

**Study size:** a single population proportion formular [12] was used in calculating the sample size with the following: 20% as prevalence of disability [13], a confidence level of 95%, 5% margin of error and allowing a 10 % non-response rate.

**Sampling technique:** a total of 340 respondents infected with onchocerciasis were selected using a two-stage sampling technique. In the first stage, seven out of the seventeen local government areas (LGAs) were selected using a simple random sampling technique by balloting. In the second stage, from a list of all the wards in the selected 7 LGAs, two wards were selected in each LGA using a simple random sampling technique through balloting. Thus, fourteen wards were selected in the 7 local government areas. In the selected wards, consecutive recruitment of persons with onchocerciasis was done. This was achieved with the assistance of health workers in the onchocerciasis unit of the State Ministry of Health who helped with the identification of onchocerciasis lesions through physical examinations of the respondents.

**Data collection instruments and measurements:** the level of disability in persons infected with onchocerciasis was assessed using the WHO Disability Assessment Schedule 2.0 (WHODAS 2.0) tool which is a 36-item questionnaire [14]. It assesses disability across six domains which include; cognition (understanding and communicating), mobility, self-care, getting along with people, life activities (i.e. household, work, and/or school activities), and participation in community activities. The disability scores of these 6 domains were weighted as 1=none, 2=mild, 3=moderate, 4=severe, and 5=extreme. For the overall disability scores, the weighted scores were converted to 1=20%, 2=40%, 3=60%, 4=80%, 5=100%. Furthermore, the respondents were categorized into no disability for scores <50% and some disability when they scored ≥50%. The WHO Model Disability Survey (MDS) questionnaire [15] was adapted and used to assess the perception of the attitude of society towards people affected with onchocerciasis and the perception of being a burden among respondents with onchocerciasis. The overall perception of the attitude of society towards onchocerciasis patients was categorized into poor perception for scores <50% and ≥50% for good perception. The perception of being a burden to the people was assessed by “Yes” or “No” response to whether the respondent considers himself/herself as a burden to others.

**Data analysis:** data entry and analysis were done using IBM Statistical Package for Social Sciences (SPSS), version 25. Continuous variables were summarized using mean and standard deviation, while categorical variables were summarized using frequencies and proportions. The outcome measure of the study was the proportion of respondents who had a disability. The Chi-square test was used to measure associations between independent categorical variables and level of disability. Variables with a p-value <0.2 at Chi-square analysis were subjected to logistic regression analysis to determine predictors of disability. The level of statistical significance was set at a p-value <0.05 for all inferential analyses; the results of the binary logistic regression analysis were presented using an adjusted odds ratio and 95% confidence interval. The socio-economic status index of the respondents was developed using Principal Component Analysis, (PCA). The input to the PCA included information on the type of housing, ownership of fifteen household items that included having electric power, radio, refrigerator, functional car, television, mobile phone, gas cooker, bicycle, kerosene, generator, rechargeable lamp, electric iron, electric fan and air conditioner, the principal type of toilet facility, the main source of energy, household consumption of food and non-food items and average monthly income of respondent and that of his/her spouse where applicable [16]. These were used to develop the wealth index according to quartiles. The quartiles included Q1 = Poorest, Q2= The Very Poor, Q3= The Poor, and Q4= The Least Poor. The quartiles were further dichotomized into low socio-economic class comprising the poorest and very poor and high socio-economic class made up of the poor and least poor groups.

**Availability of data and materials:** the datasets generated and/or analysed during the study are not publicly available due to confidentiality policies but are available from the corresponding author on reasonable request

**Ethical consideration:** the study was conducted in compliance with the ethical guidelines of the Health Research Ethics Committee of the University of Nigeria Teaching Hospital, Enugu after obtaining ethical approval (reference number; UNTH/CSA/329/VOL.5). The respondents gave written informed consent after they had been duly informed of the purpose of the study and assured of the confidentiality of volunteered information.

## Results

**Socio-demographic characteristics of the respondents:** in table 1, the mean age of the respondents was 46.8±17.5 years. The highest proportion of the respondents, 35.6% were 55 years and above while the least proportion, 15.9% were between 35 and 40 years. Majority of the respondents, 58.5% were females. The highest proportion of the respondents, 64.4% were self-employed while the least proportion, 16.2% were on salaried employment.

**Table 1:** socio-demographic characteristics of respondents with onchocerciasis

Variables	Frequency (n=340)	Percent (%)
**Age groups (years)**		
<35	96	28.2
35-44	54	15.9
45-54	69	20.3
≥55	121	35.6
Mean±SD	46.8±17.5	
**Gender**		
Male	141	41.5
Female	199	58.5
**Educational attainment**		
No formal education	66	19.4
Primary education	89	26.6
Secondary education	126	37.1
Tertiary education	59	17.4
**Marital status**		
Never married	81	23.8
Married	242	71.2
Separated/divorced	7	2.1
Widowed	10	2.9
**Employment status**		
Unemployed	66	19.4
Self-employed	219	64.4
Salaried employment	55	16.2
**Socio-economic class**		
Low socio-economic class	170	50.0
High socio-economic class	170	50.0

**Level of disability among the respondents in the six disability domains:** the disability domains mainly affected included getting along with other people (61.8%) and participation in community activities (54.1%) while the least affected were self-care (26.2%) and life activities including domestic responsibilities (45.0%), (Table 2). Regarding the prevalence of disability, 259 (76.2%) respondents had some disability while 81 (23.8%) had no disability.

**Table 2:** level of disability among the respondents in the 6 disability domains

Variable	Mean±SD	None n (%)	Mild n (%)	Moderate n (%)	Severe n (%)	Extreme n (%)
Domain 1 Cognition	1.5±0.8	165(48.5)	111(32.6)	42(12.4)	17 (5.0)	5 (1.5)
Domain 2 Mobility	1.6±0.9	165(48.5)	101(29.7)	47(13.8)	18 (5.3)	9 (2.6)
Domain 3 Self-care	1.2±0.5	251(73.8)	75 (22.1)	9 (2.5)	3 (0.9)	2 (0.6)
Domain 4 Getting along	1.6±0.8	130(38.2)	144(42.4)	48 (14.1)	13 (3.8)	5 (1.5)
Domain 5 Life activities	1.7±1.0	187 (55.0)	97 (28.5)	32 (9.4)	24 (7.1)	0 (0.0)
Domain 6 Participation	1.5±0.7	156 (45.9)	125(36.8)	44(12.9)	10 (2.9)	5 (1.5)
Total WHODAS 2.0 score	1.5±0.7	81 (23.8)	205(60.3)	36(10.6)	15 (4.4)	3 (0.9)

**Factors affecting disability among persons with onchocerciasis:** in bivariate analysis, age, educational attainment, socioeconomic status, employment status, perception of attitude to others and perception of being a burden to people were significantly associated with disability. On further analysis, age became a predictor of disability. The respondents who were less than 48 years old were five times less likely to have a disability when compared with those who were 48 years and above, (AOR=0.2, CI=0.1-0.4, p<0.001), (Table 3).

**Table 3:** factors affecting disability among respondents with onchocerciasis

Variable	Disability (n=340)		χ^2^(p-value on bivariate analysis)	AOR in multivariate analysis (95% CI)
	Yes N (%)	No N (%)		
**Age of respondents**				
<48 years	113 (64.2)	63 (35.8)	28.817 (<0.001)	0.2 (0.1–0.4)
≥48 years	146 (89.0)	18 (11.0)		1
**Gender**				
Male	103 (73.0)	38 (27.0)	1.298 (0.255)	NA
Female	156 (78.4)	43 (21.6)		
**Educational attainment**				
No formal education	57 (86.4)	9 (13.6)	10.011 (0.018)	1.8 (0.6–5.3)
Primary education	70 (78.7)	19 (21.3)		1.4 (0.5–3.4)
Secondary education	95 (75.4)	31 (24.6)		1.6 (0.7–3.5)
Tertiary education	37 (62.7)	22 (37.3)		1
**Marital status**				
Married	189 (78.1)	53 (21.9)	1.710 (0.191)	1.1 (0.6–2.1)
Single**	70 (71.4)	28 (28.6)		1
**Socio-economic class**				
Low socio-economic class	138 (81.2)	32 (18.8)	4.684 (0.030)	0.9 (0.5–1.7)
High socio-economic class	121 (71.2)	49 (28.8)		1
**Employment status**				
Unemployed	47 (71.2)	19 (28.8)	6.489 (0.039)	1.2 (0.5–3.2)
Self-employed	176 (80.4)	43 (19.6)		1.4 (0.6–3.2)
Salaried employed	36 (65.5)	19 (32.5)		1
**Perception of attitude to others**				
Good	196 (73.1)	72 (26.9)	6.454 (0.011)	0.5 (0.2–1.0)
Poor	63 (87.5)	9 (12.5)		1
**Perception of being a burden to people**				
Yes	29 (96.7)	1 (3.3)	7.512 (0.006)	6.4 (0.8–50.0)
No	230 (74.2)	80 (25.8)		1

## Discussion

The clinical manifestations among people with onchocerciasis usually present with long-term complications due to late detection of this disease thus affecting their ability to carry out their daily activities or participate in society [6]. This ultimately may lead to disability [8]. A high prevalence of disability among persons with onchocerciasis was observed in this study and being in an older age group was a major determinant of disability in the individuals.

In this study, 76.2% of the respondents had some disability while 23.8% had no disability. Although disability rates in onchocerciasis are under-reported, similar studies relating to other NTDs such as leprosy, lymphatic filariasis and podoconiosis have reported some level of disability among their respondents [9, 17, 18]. This high prevalence of disability noted among people with onchocerciasis is probably due to late detection and poor treatment of the disease [8]. Furthermore, the high prevalence of disability reported in this study and previous studies [9, 17, 18] may be linked to the rural setting where most of the participants resided. In these areas, patients may not have the right information to seek health care at an early stage of the disease due to poor access to print and electronic media where health promotion programs are readily available [19]. All of these predispose persons with onchocerciasis to poor detection and treatment of the disease which ultimately leads to disability.

The disability domains mainly affected included getting along with other people (61.8%) and participation in community activities (54.1%). The findings observed in this study may be because onchocerciasis causes visual impairments and body disfigurements which predisposes the affected individuals to depression, stigmatization and social exclusions [8]. These findings imply that onchocerciasis being a cause of disability, is of public health importance, as it interferes negatively with people´s lives including their participation in community activities and this can affect their mental health leading to poor quality of life [6]. Also, due to not getting along with people, some farmers in the study area may vacate their fertile lands for unfertile areas, leading to poor harvest, lack of food security among households and poverty.

Furthermore, environmental and personal factors play a key role in the level of disability among persons with onchocerciasis. In this study, the respondents who were less than 48 years were 5 times less likely to have a disability when compared with those aged 48 years and above. The finding agrees with that of a study in Ethiopia [20] which revealed an increase in the prevalence of disability in the older age group. The probable explanation for this may be that onchocerciasis being a neglected disease that is not diagnosed on time for immediate care in affected people leads to a longer duration of symptoms and complications [5]. Thus the older age groups may have been unknowingly affected for a prolonged time which predisposes them to long-term consequences of the disease and a greater risk of disability [20]. Also, the finding may be linked to the certainty that aging is associated with chronic degenerative diseases that worsen participation in daily life activities, especially among older people with onchocerciasis leading to an increase in disability and poor well-being [21].

**Limitation of the study:** the limitation of this study is that the information obtained from the respondents could be subjective due to the possibility of recall bias and the self-reporting nature of the study instrument. Perhaps, a complimenting qualitative method will help to obtain an in-depth understanding of the individual experiences.

## Conclusion

Disability is a usual occurrence among people with onchocerciasis and the commonest disability domains affected were restriction in participating in community activities and difficulty in getting along with other people. Therefore, stakeholders should strengthen the community awareness program on onchocerciasis to relieve the stigma associated with getting along with people or participation in community activities and to ensure early detection of disease to prevent disability. Also, community health care workers should routinely screen the affected individuals for any impairments to ensure early detection of the disease-associated disability, especially in the older age group.

### What is known about this topic

- Onchocerciasis is a neglected tropical disease that causes debilitating skin and ocular diseases which may predispose the affected individuals to disability;
- People with disabilities due to physical or visual impairments attributed to neglected tropical diseases face several challenges which may affect their social and mental health.

### What this study adds

- The study fills the information gap on the prevalence of disability in persons with onchocerciasis;
- The disability domains commonly affected were restrictions in participating in community activities and difficulty in getting along with other people;
- Being in the older age group was a major determinant of disability among persons with onchocerciasis.
